# Mental Health Distress and Related Factors Among Prefectural Public Servants Seven Months After the Great East Japan Earthquake

**DOI:** 10.2188/jea.JE20130138

**Published:** 2014-07-05

**Authors:** Yuriko Suzuki, Maiko Fukasawa, Akiko Obara, Yoshiharu Kim

**Affiliations:** 1 Department of Adult Mental Health, National Institute of Mental Health, National Center of Neurology and Psychiatry, Kodaira, Tokyo, Japan; 2 Miyagi Mental Health and Welfare Center, Osaki, Miyagi, Japan; 3 National Information Center of Disaster Mental Health, National Institute of Mental Health, National Center of Neurology and Psychiatry, Kodaira, Tokyo, Japan

**Keywords:** mental health, disaster, worker, cross-sectional study, risk factors

## Abstract

**Background:**

To develop an empirically informed support measure for workers, we examined mental health distress and its risk factors among prefectural public servants who were affected by the Great East Japan Earthquake and faced a demanding workload in the midterm of the disaster.

**Methods:**

We conducted a self-administered health survey of all public servants in the Miyagi prefectural government two and seven months after the Great East Japan Earthquake (3743 workers, 70.6% of all employees). We calculated odds ratios (ORs) and 95% confidence intervals (CIs) for mental distress (defined as K6 score ≥10) in the domain of disaster-work-related stressors, work-related stressors, and disaster-related stressors.

**Results:**

Among those with better levels of workplace communication, the only factor that increased the risk of mental distress was not taking a non-work day each week (adjusted OR 2.55, 95% CI 1.27–5.14). Among those with poorer levels of workplace communication, in addition to not taking a non-work day each week (adjusted OR 3.93, 95% CI 3.00–5.15), handling residents’ complaints (adjusted OR 1.55, 95% CI 1.00–2.42), having dead or missing family members (adjusted OR 2.87, 95% CI 1.53–5.38), and living in a shelter more than two months after the disaster (adjusted OR 2.80, 95% CI 1.32–5.95) increased the risk of mental distress.

**Conclusions:**

All workers should be encouraged to take a non-work day each week. Among workers with poor workplace communication, special attention should be given to those who handle residents’ complaints, have lost a family member(s), and are living in a shelter for a prolonged period of time.

## INTRODUCTION

The Great East Japan Earthquake hit northern Japan on March 11, 2011, causing one of Japan’s worst natural disasters. This disaster strained the mental health of not only residents^1^ but also public servants. Recently, workers have become an important target of post-disaster health monitoring because they are key to the recovery of local governmental entities and, therefore, communities.^2^ In the case of the Great East Japan Earthquake, based on first-hand experience, the mental health community warned that public servants in the affected areas faced increased levels of stress in the aftermath of the disaster.^3^^,^^4^ In response to these clinical observations, the Japanese Society of Neurology and Psychiatry issued an emergency statement to protect the health of public servants (i.e., people working for prefectures and municipalities).^5^ Given their extensive experience responding to natural disasters, mental health professionals were concerned about public servants’ mental and physical health. Japan’s fairly strong administrative structure, with its central and prefectural governments, has municipalities serving as the service point for local residents.^6^ Public servants play a major role in the immediate response and recovery phases of a disaster and, unlike transient rescue workers who are deployed to an affected area for a defined period of time, face multiple stressors as victims of the disaster as they have a high workload in the disaster’s aftermath and are local residents living in the disaster-affected community.

Past research has revealed the importance of critical stress management of rescue workers such as fire fighters, police, ambulance personnel, and unaffiliated volunteers.^7^^,^^8^ Rescue workers’ stress is conventionally categorized as (1) basic stress, which is increased by changes in one’s day-to-day environment when workers are faced with, for example, being away from family without adequate communication, working with new people from different cultures, working in uncertain conditions, and having to assimilate new information; (2) cumulative stress, composed of various stress factors such as a heavy workload, poor communication, the frustration of not being able to meet the beneficiaries’ needs, having to cope with situations in which one feels powerless, lack of basic comforts, and inability to rest or relax; and (3) traumatic stress or critical incident stress due to life-threatening events such as witnessing the death of a coworker, or seeing a dead body or other dreadful situations.^9^ Such risk factors are scrutinized among workers likely to experience critical stress, the primary outcome of which is traumatic stress.^10^ However, empirical evidence on disaster work-related stress in public servants has not yet been fully examined. Close examination of these public servants will help determine how to support them in times of disaster because such workers have various known job stressors, such as overwork and quality of communication, during ordinary times.

In the prefectural government of Miyagi, Japan, a comprehensive measure to monitor and support the health status of workers was put in place after the Great East Japan Earthquake. Utilizing this framework, this study was designed to disentangle multiple stressors in the midterm of the disaster by examining the relationship between mental health status and multifaceted stressors, and to empirically guide a strategy to promote the mental health and readiness of workers. Thus, the study aimed to identify the disaster- and work-related stressors and mental health status of prefectural government public servants who experienced the Great East Japan Earthquake and subsequent increase in workload. We hypothesized that disaster-related work stressors, work-related stressors, or disaster-related stressors might affect the mental health of public servants differently, depending on the level of known work-related stress and quality of workplace communication.

## METHODS

### Study design

We conducted two cross-sectional studies within one year of the Great East Japan Earthquake. We collected data two and seven months after the earthquake (May 2011 and October 2011). The study design is a cross-sectional study of mental health outcomes after seven months to investigate possible risk factors derived at two time points depending on the accuracy and availability of such survey information.

### Participants

All public servants in Miyagi Prefecture, Japan, were the target of the surveys (*n* = 5305), conducted as part of a health program run by the Prefectural Labor Welfare Division. Miyagi prefecture is the closest prefecture to the epicenter of the Great East Japan Earthquake. The number of dead and missing due to the disaster exceeded 10 000,^11^ the largest death toll among the prefectures affected by the disaster.

### Procedure

We invited all public servants in the Miyagi prefectural government to participate in a web-based survey as part of a health prevention and promotion program run by the Prefectural Labor Welfare Division. Participants were directed to a self-administered questionnaire primarily to self-monitor their health status, with special attention paid to work pressures after the Great East Japan Earthquake. At the end of the survey, information was provided to participants about mental health resources, and they were invited to an appointment with a psychologist, psychiatrist, and occupational physician.

### Variables

We measured non-specific mental health distress seven months after the disaster as a primary outcome using Kessler’s K6 scale.^12^ In the K6 scale, participants were asked if they had the following symptoms during the past 30 days: feeling so sad that nothing could cheer you up, nervous, hopeless, restless or fidgety, that everything was an effort, or worthless. Each question was rated on a 5-point Likert scale from zero (none of the time) to four (all of the time), with higher scores signifying worse mental health status (range: 0–24). The Japanese version of the K6 has been validated.^13^^,^^14^

The health survey questionnaire elicited the following information: (1) basic information, including age, gender in May 2011, working department, and degree of involvement in disaster-related work (not at all to most of the time, 5-point Likert scale) in October 2011; and (2) possible risk factors in the following domains:

1. **Domain of disaster-related work stressors**. Solicited in the May 2011 survey, these stressors are specifically related to disaster work, working at a disaster-area work site (coastal or inland area), working at a morgue, and handling residents’ complaints.

2. **Domain of work-related stressors**. These are work-related stressors that are reported even in typical work settings, with the October 2011 survey asking questions including the amount of overtime assessed by the longest hours worked since the disaster and during the previous month (i.e., September 2011), whether or not a non-work day was taken each week, and the May 2011 survey asking questions including the level of workplace communication by rating the quality of communication (poor, reasonable, or good) between bosses, colleagues, and subordinates.

3. **Domain of disaster-related stressors**. These stressors specifically relate to disaster victims, with questions in the May 2011 survey concerning whether they had dead or missing family members and if they lived someplace other than in their own house (e.g., in a shelter as of May 2011), and questions in the October 2011 survey asking about the degree of property damage reported in the official damage report (i.e., total collapse, mostly collapsed, half-collapsed, partial collapse, none).

### Ethical standards

All procedures contributing to this work comply with the ethical standards of the relevant national and institutional committees on human experimentation and with the Helsinki Declaration of 1975, as revised in 2008. The study protocol was approved by the institutional review board of the National Center of Neurology and Psychiatry.

### Statistical analysis

Among all workers, 4334 completed the survey in May 2011, and 4413 completed the survey in October 2011. We analyzed the dataset of those who participated in both surveys (3743 workers, 70.6% of all workers).

First, we grouped those who scored ≥10 on the K6 scale in October 2011, which comprised the upper 10% of participants, indicating that they had mental distress. The upper 10% was conservatively chosen because approximately 10% of the population is estimated to have a mild level of mental disorder regardless of disaster exposure.^15^ Also, the World Mental Health Survey in Japan revealed that 8.8% of community residents at any given time will have had some type of mental disorder during the past 12 months.^16^ Next, we used two-tailed chi-square tests to examine associations between mental distress and exposure variables. We used K6 scale outcomes as the dependent variable, and exposure variables in disaster-related work stressors, work-related stressors, or disaster-related stressors as independent variables. We adjusted for age group (18–29, 30–39, 40–49, and 50–65 years), gender, and degree of involvement in disaster-related work at a time in the multivariate analysis, as those variables were associated with mental distress in the bivariate analysis. We chose to control for the degree of involvement in disaster-related work based on the assumption that greater involvement in this type of work leads to greater burden of mental distress. We were also interested in identifying other modifiable risk factors in the workplace after the disaster.

Poor communication with coworkers also reportedly increases the risk of work-related mental health in ordinary times,^17^ and the bivariate analysis indicated that level of workplace communication was associated with mental distress (Table 2). Because the level of workplace communication is not necessarily a disaster-related stressor, we specifically chose to include as factors of interest, the disaster-related risk factors in the multivariate analysis for separate groups based on quality of communication (good, reasonable, or poor) in order to control for the known workplace risk factor of poor workplace communication.

**Table 2.** Relationship between potential stressors on participants’ mental health status as measured by the K6 scale

	All		K6 < 10		K6 ≥ 10		*df*	χ^2^	*P* value
		
	*n*	%	*n*	%	*n*	%			
Disaster-work-related stressors									
Working at a disaster-area work site (*n* = 3743)									
Inland area	3164	84.5	2867	84.7	297	83.0	1	0.7	0.388
Coastal area	579	15.5	518	15.3	61	17.0			
Working at a morgue (*n* = 3739)									
No	3488	93.3	3152	93.2	336	93.9	1	0.2	0.652
Yes	251	6.7	229	6.8	22	6.2			
Handling residents’ complaints (*n* = 3739)									
No	3492	93.4	3167	93.7	325	90.8	1	4.4	0.036
Yes	247	6.6	214	6.3	33	9.2			
Work-related stressors									
Working overtime during the previous month (*n* = 3682)									
Less than 20 hours	2771	75.3	2538	76.2	233	66.0	2	32.5	<0.001
20 to 40 hours	614	16.7	549	16.5	65	18.4			
More than 40 hours	297	8.1	242	7.3	55	15.6			
Working overtime in the month with the longest hours worked (*n* = 3673)									
Less than 40 hours	2113	57.5	1941	58.5	172	48.9	2	12.3	0.002
40 to 80 hours	942	25.7	830	25.0	112	31.8			
More than 80 hours	618	16.8	550	16.6	68	19.3			
Workplace communication (*n* = 3742)									
Poor	102	2.7	63	1.9	39	10.9	2	116.5	<0.001
Reasonable	2693	72.0	2425	71.7	268	74.9			
Good	947	25.3	896	26.5	51	14.3			
Workplace communication (*n* = 3742) (summarized)									
Good	947	25.3	896	26.5	51	14.3	1	25.6	<0.001
Poor and reasonable	2795	74.7	2488	73.5	307	85.8			
Took a non-work day each week (*n* = 3739)									
Yes	2768	74.0	2611	77.2	157	44.0	1	185.4	<0.001
No	971	26.0	771	22.8	200	56.0			
Disaster-related stressors									
Dead or missing family members (*n* = 3742)									
No	3648	97.5	3308	97.7	340	95.2	1	8.2	0.004
Yes	94	2.5	77	2.3	17	4.8			
Property damage (*n* = 3741)									
Less than half-collapse	3199	85.5	2912	86.1	287	80.2	1	18.9	0.005
Half-collapse or worse	542	14.5	471	13.9	71	19.8			
Living someplace other than in their own house (eg, shelter) (*n* = 3740)									
No	2894	77.4	2647	78.2	247	69.4	2	18.9	<0.001
Previously, yes	776	20.8	681	20.1	95	26.7			
Currently, yes (as of May, 2011)	70	1.9	56	1.7	14	3.9			

Chi-square tests were used.

All statistics were generated using Stata 12.0 for Windows (StataCorp LP, College Station, TX, USA). Statistical significance was set at 0.05, and all tests were two-tailed.

## RESULTS

Table 1 shows the basic characteristics of the study participants. Those with a K6 score ≥10 numbered 358 (9.6%). For reference, using two cutoffs widely used in ordinary times, 1224 (32.7%) workers scored ≥5 and 164 (4.4%) scored ≥13. Regarding gender, age group, and degree of involvement in disaster-related work, those with mental distress (K6 score ≥10) were significantly more likely to be female, in younger age groups, and more involved in disaster-related work.

**Table 1.** Basic characteristics of the participants in relation to mental health status as measured by the K6 scale (*n* = 3743)

	All		K6 < 10		K6 ≥ 10		*df*	χ^2^/*t*	*P* value
		
	*n*	%	*n*	%	*n*	%			
	3743		3385		358				
Gender									
Male	2903	77.6	2644	78.1	259	72.4	1	6.2	0.013
Female	840	22.4	741	21.9	99	27.7			
Age group (years)									
18–29	445	11.9	392	11.6	53	14.8	3	29.3	<0.001
30–39	898	24.0	795	23.5	103	28.8			
40–49	1255	33.5	1118	33.0	137	38.3			
50–65	1145	30.6	1080	31.9	65	18.2			
Mean age (standard deviation)	43.2	(10.4)	43.5	(10.4)	40.7	(9.4)	3741	4.8	<0.001
Department									
General affairs	510	13.6	468	13.8	42	11.7	9	6.7	0.671
Earthquake reconstruction and planning	127	3.4	120	3.6	7	2.0			
Living environment	222	5.9	196	5.8	26	7.3			
Health and welfare	658	17.6	586	17.3	72	20.1			
Economic chamber of commerce and tourism	742	19.8	676	20.0	66	18.4			
Agriculture, forestry and fisheries	575	15.4	519	15.3	56	15.6			
Civil section	657	17.6	594	17.6	63	17.6			
Teller stations	68	1.8	61	1.8	7	2.0			
Public business administration	65	1.7	58	1.7	7	2.0			
Others	119	3.2	107	3.2	12	3.4			
Degree of involvement in disaster-related work									
Disaster-related work as primary work	349	9.3	313	9.3	36	10.1	4	17.7	0.001
Mainly disaster-related work	422	11.3	360	10.7	62	17.4			
About the same as primary work	492	13.2	441	13.1	51	14.3			
Mainly primary work	1103	29.5	1014	30.0	89	25.0			
Not involved	1369	36.7	1251	37.0	118	33.2			

Chi-square tests or *t*-tests were used.

Table 2 shows the bivariate analysis between mental health status and possible stressors. We found disproportionately more mental distress as measured by the K6 scale at a statistically significant level in those who handled residents’ complaints, worked overtime both during the month of the longest hours worked and during the month prior to the second survey, reported lower levels of workplace communication, did not take a non-work day each week, had dead or missing family members, faced more severe property damage (half-collapse or worse), and were living in a shelter two months after the disaster (i.e., as of May 2011).

Based on multivariate analysis, Table 3 shows adjusted ORs and 95% CIs of possible risk factors shown in the aforementioned bivariate analysis. Results revealed that the following risk factors significantly increased risk of mental distress: a lower level of workplace communication (adjusted OR 1.97, 95% CI 1.43–2.71), not taking a non-work day each week (adjusted OR 3.95, 95% CI 3.08–5.07), having dead or missing family members (adjusted OR 2.23, 95% CI 1.23–4.03), and living in a shelter as of May 2011 (adjusted OR 2.55, 95% CI 1.27–5.14).

**Table 3.** Adjusted odds ratios and 95% confidence intervals of disaster-work-related, work-related, and disaster-related stressors on participants’ mental health distress (K6 score ≥ 10)^a^ by quality of workplace communication^b^

	All (*n* = 3666)			Workplace communication					

				Good (*n* = 908)			Poor and reasonable (*n* = 2732)		
		
	*OR*	95% CI	*P* value	*OR*	95% CI	*P* value	*OR*	95% CI	*P* value
Gender (reference: Male)									
Female	1.44	1.08–1.91	0.012	1.42	0.65–3.10	0.374	1.42	1.93–1.04	0.025
Age group (reference: 18–29 year old)									
30–39 year old	0.89	0.61–1.29	0.526	0.77	0.31–1.94	0.584	0.90	1.37–0.60	0.630
40–49 year old	0.75	0.52–1.08	0.116	0.48	0.18–1.24	0.128	0.80	1.20–0.54	0.282
50–65 year old	0.48	0.32–0.73	0.001	0.71	0.27–1.85	0.487	0.45	0.72–0.28	0.001
Degree of involvement in disaster related work (reference: Not involved)									
Mainly primary work	1.02	0.75–1.38	0.898	1.04	0.41–2.63	0.939	1.02	1.41–0.74	0.906
About the same as primary work	1.07	0.73–1.57	0.712	2.14	0.80–5.71	0.130	0.95	1.45–0.63	0.823
Mainly disaster related work	1.48	1.01–2.18	0.045	3.17	1.22–8.22	0.018	1.27	1.94–0.82	0.284
Disaster related work as primary work	1.15	0.74–1.77	0.533	2.61	0.93–7.38	0.069	0.99	1.61–0.61	0.966
Disaster-related work stressor									
Work site (reference: Inland area)									
Coastal area	0.93	0.67–1.28	0.657	0.54	0.20–1.46	0.223	1.01	0.72–1.43	0.954
Work at a morgue (reference: No)									
Yes	1.08	0.67–1.74	0.757	0.76	0.17–3.34	0.718	1.11	0.67–1.84	0.692
Complaint handling by residents (reference: No)									
Yes	1.48	0.98–2.22	0.061	0.98	0.32–2.97	0.968	1.55	1.00–2.42	0.050
Work related stressor									
Working overtime during the previous month (reference: Less than 20 hours)									
20 to 40 hours	0.74	0.53–1.04	0.081	0.71	0.29–1.73	0.456	0.75	0.53–1.08	0.127
More than 40 hours	1.04	0.69–1.57	0.834	0.94	0.34–2.62	0.911	1.08	0.69–1.69	0.740
Working overtime in the month longest hour worked (reference: Less than 40 hours)									
40 to 80 hours	1.23	0.92–1.65	0.158	1.15	0.53–2.48	0.720	1.25	0.91–1.71	0.170
More than 80 hours	1.07	0.75–1.54	0.707	0.92	0.37–2.27	0.857	1.11	0.75–1.64	0.614
Communication at work (reference: Good)									
Poor and reasonable	1.97	1.43–2.71	<0.001	—	—		—	—	
A holiday a week (reference: Yes)									
No	3.95	3.08–5.07	<0.001	3.71	1.92–7.18	<0.001	3.93	3.00–5.15	<0.001
Disaster related stressor									
Death or missing of family members (reference: No)									
Yes	2.23	1.23–4.03	0.008	—	—		2.87	1.53–5.38	0.001
House damage (reference: Less than half-collapse)									
Half-collapse or severer	1.27	0.93–1.75	0.134	0.87	0.33–2.26	0.769	1.32	0.94–1.86	0.106
Living in other than their house (eg, shelter) (reference: No)									
Previously yes	1.25	0.95–1.64	0.115	0.94	0.43–2.07	0.885	1.32	0.99–1.78	0.062
Currently yes (as of May, 2011)	2.55	1.27–5.14	0.009	1.97	0.20–18.92	0.559	2.80	1.32–5.95	0.007

OR = odds ratio, CI = confidence interval.

^a^Adjusted by gender, age group, and degree of involvement in disaster-related work.

^b^Answers regarding communication level were missing for 26 participants, and no imputation was done. Thus, the total number of participants in the good communication and poor and reasonable groups (*n* = 908, *n* = 2732, respectively) was not equal to the number of all participants (*n* = 3666).

In the analysis stratified by different levels of communication, among those who had good levels of workplace communication, not taking a non-work day each week (adjusted OR 3.71, 95% CI 1.92–7.18) was the only factor that increased the risk of mental distress. Among those who had poor or reasonable workplace communication, in addition to not taking a non-work day each week (adjusted OR 3.93, 95% CI 3.00–5.15), handling residents’ complaints (adjusted OR 1.55, 95% CI 1.00–2.42), having dead or missing family members (adjusted OR 2.87, 95% CI 1.53–5.38), and living in a shelter as of May 2011 (adjusted OR 2.80, 95% CI 1.32–5.95) increased the odds of mental distress among prefectural government workers.

## DISCUSSION

In this study, mental distress was identified in 358 workers (9.6%) in the Miyagi prefectural government who were in the upper 10% on the K6 scale (score ≥10). In 2009, 11.5% of Miyagi residents scored ≥10, and in 2006, 12.1% of them did so.^18^ A previous report on municipal public servants showed that the proportion of workers with mental distress (K6 score ≥9) was 8.2% in ordinary times.^19^ The percentage in the present study fell in between the percentages reported in these previous studies. One explanation is the different population composition, as our participants were predominantly male, middle-aged, working people compared to the community studies’ participants, who were predominantly non-working elderly people. Moreover, although government workers were affected by the disaster, their jobs were somewhat secure even after the disaster compared to those of community residents who faced less job security as a consequence of the disaster. Socioeconomic factors, including job security, are known associated factors for mental distress during times of disaster as well as in ordinary times.^20^^,^^21^ Under these circumstances, timing the survey seven months after the disaster might have allowed for recovery to levels of mental distress seen in ordinary times among the workers.

Comparing the results of bivariate and multivariate analyses in regards to handling residents’ complaints, working overtime during the previous month, and working overtime in the month with the longest hours worked, no statistical significance was observed in the multivariate analysis for all participants. The strong associations between degree of involvement in disaster-related work and these variables were observed in bivariate analyses (results available upon request), and no significance was seen after controlling for the degree of involvement in disaster-related work. Similarly, because property damage and living someplace other than in their own house (eg, a shelter) were associated in the bivariate analysis, statistical significance was not observed after controlling for the variables in the multivariate analysis.

### Risk factors

In examining the association between possible risk factors and mental distress, the work-related stressors of having poor or reasonable levels of workplace communication and not taking a non-work day each week, as well as the disaster-related stressors of having dead or missing family members and living in a shelter as of May 2011, increased the risk of mental distress in the analysis of all workers. Disaster-related stressors that are often reported as risk factors, such as working at a morgue and handling residents’ complaints, were not associated with mental distress in the overall analysis.

Work-related stressors (eg, poor workplace communication or insufficient rest) had a significant impact on mental distress, especially in times of disaster. Even in ordinary times, work-related stress among workers in local governments is an area of concern, along with stringent budgets and pressing human resource needs.^6^ In prefectures and municipalities within the affected area, temporary officers were employed to fill the gap between decreased human resources and a demanding workload in the affected area. These measures are empirically supported in view of maintaining the mental health of workers. The importance of workplace communication is supported by a study on industrial mental health in ordinary times,^17^ and workplace communication remains important in times of disaster. We added a separate analysis stratified by level of communication and found that the disaster-related stressors of having dead or missing family members and living in a shelter as of May 2011 (signifying prolonged unstable residence two months after the disaster) increased the risk of mental distress. This supports the findings of a previous study, where the loss of loved ones and the day-to-day stresses related to evacuation were shown to be associated with worsened mental health status.^22^

In the analysis stratified by level of communication, not taking a non-work day each week was the only significant risk factor among those who reported having good workplace communication. On the other hand, handling residents’ complaints, having dead or missing family members, and living in a shelter as of May 2011 increased the risk of mental distress among those who reported poor or reasonable levels of workplace communication. Unlike the results for those with a poor or reasonable level of workplace communication, we could not obtain results on the association between family loss and mental distress for participants with good workplace communication because no one in this category had experienced family loss. By design, we were not able to examine the causal relationship between family loss and mental health, and further investigation is needed. Results of the analysis stratified by level of workplace communication should be interpreted with caution because the measure of communication level is a subjective single-item question that is not validated. Bearing this limitation in mind, these findings suggest that for all workers, regardless of their quality of workplace communication, having at least a non-work day each week is strongly recommended. In a disaster setting, although this is easier said than done, supervisors must understand the importance of having at least one non-work day per week and make organizational efforts to secure holidays for staff. For workers with a poor or reasonable level of workplace communication, offering them an opportunity to share their concerns with coworkers might help lessen the psychological burden of handling residents’ complaints and dealing with grief, loss, and the inconvenience of evacuation. Also, these workers might benefit from organizational measures that accommodate the work environment, such as work rotations and buddy systems. Social support is known to be one of the strongest protective factors of mental health after a disaster.^23^ Among workers, coworkers are the most accessible source of social support, and thus fostering quality workplace communication is key to maintaining mental health at work.

### Limitations

The survey response rate was fairly high because it was conducted in an occupational health program setting. Workers were identifiable under the prefectural governments’ internet-communication system. Although we informed workers that the survey results would be concealed and independent of any performance evaluation, they might have felt pressured to respond to the questionnaires. Thus, the possibility of under-reporting of mental distress remains. Further, approximately 5% of the workers did not respond to either of the surveys even in the abovementioned context. Thus, it is possible that those who were too busy to respond might also have been those with the highest needs, although we could not confirm this speculation without data on nonresponders’ characteristics.

Second, previous studies investigating workers’ post-disaster mental health mainly focused on traumatic reactions and critical stress.^24^ In this study, however, given the nature and extent of stressors among public servants, we chose general mental health status as measured by the K6 scale as a main outcome. Thus, the presentation of reactions and their associated risk factors might have differed from that of traumatic reactions. Future research is needed on outcomes of traumatic stress among public servants in times of disaster.

Third, we did not examine other known mental health risk factors such as smoking, alcohol consumption, or chronic health conditions because they fell outside the focus of this analysis. In addition, preexisting mental disorders consistently increase the risk of mental distress in times of disaster; however, we did not consider this factor because this information could not be obtained. Although available, we did not include mental distress as of May 2011 because mental health in the early phase of a disaster does not necessarily reflect a person’s baseline level of mental health.^25^

Finally, this survey included all public servants in the Miyagi prefectural government; therefore, it included workers who were not involved in disaster-related work based on their assigned jobs and work locations. We found that 20.6% of all participants were involved in disaster-related work as primary work and mainly handled disaster-related work; thus, not all participants should be assumed to have been directly affected by the disaster. Bearing this assumption in mind, those with a lesser degree of disaster involvement can serve as comparisons by controlling for level of involvement in disaster-related work. Further, under the Japanese administrative structure, municipal officers are more directly involved in disaster-related work, and they have first-hand disaster experience as survivors, especially those based in coastal areas. The results of this study might not be generalizable to such workers in local municipalities. However, these results are an important reference of mental health status and its risk factors among prefectural public servants in the area affected by the Great East Japan Earthquake.

In conclusion, public servants in the affected area were both victims and workers who had to respond to extraordinary disaster-related demands. To evaluate their needs and develop empirically based measures to support them, we conducted surveys to reveal the association between mental distress and its risk factors in the domains of disaster-related-work stressors, work-related stressors, and disaster-related stressors. Overall, disaster-work-related stressors or critical stressors that are specific to a traumatic event, such as working at a morgue or handling residents’ complaints, were not associated with increased mental distress in this study population. More general approaches appropriate to an occupational health program, such as facilitating workers to take a non-work day, are needed to support workers regardless of their quality of workplace communication. Also, special attention should be paid to those who handled residents’ complaints, lost a family member(s), and lived in a shelter for a prolonged period of time, especially for workers with poor workplace communication.
